# Upper urinary tract stone disease in patients with poor performance status: active stone removal or conservative management?

**DOI:** 10.1186/s12894-017-0293-4

**Published:** 2017-11-16

**Authors:** Shimpei Yamashita, Yasuo Kohjimoto, Yasuo Hirabayashi, Takashi Iguchi, Akinori Iba, Masatoshi Higuchi, Hiroyuki Koike, Takahito Wakamiya, Satoshi Nishizawa, Isao Hara

**Affiliations:** 1 Department of Urology, Wakayama Medical University, 811-1 Kimiidera, Wakayama City, Wakayaka 641-0012 Japan; 2 Department of Urology, Hashimoto Municipal Hospital, 2-8-1 Ominedai, Hashimoto City, Wakayama 648-0005 Japan; 3 Department of Urology, Kinan Hospital, 46-70 Shinjyo, Tanabe City, Wakayama 646-8588 Japan; 4 Department of Urology, Rinku General Medical Center, 2-23 Rinkuouraikita, Izumisano City, Osaka 598-8577 Japan

**Keywords:** Poor performance status, Urolithiasis, Extracorporeal shock wave lithotripsy, Ureteroscopy, Percutaneous nephrolithotomy, Prognosis

## Abstract

**Background:**

It remains controversial as to whether active stone removal should be performed in patients with poor performance status because of their short life expectancy and perioperative risks. Our objectives were to evaluate treatment outcomes of active stone removal in patients with poor performance status and to compare life prognosis with those managed conservatively.

**Methods:**

We retrospectively reviewed 74 patients with Eastern Cooperative Oncology Group performance status 3 or 4 treated for upper urinary tract calculi at our four hospitals between January 2009 and March 2016. Patients were classified into either surgical treatment group or conservative management group based on the presence of active stone removal. Stone-free rate and perioperative complications in surgical treatment group were reviewed. In addition, we compared overall survival and stone-specific survival between the two groups. Cox proportional hazards analysis was performed to investigate predictors of overall survival and stone-specific survival.

**Results:**

Fifty-two patients (70.3%) underwent active stone removal (surgical treatment group) by extracorporeal shock wave lithotripsy (*n* = 6), ureteroscopy (*n* = 39), percutaneous nephrolithotomy (n = 6) or nephrectomy (*n* = 1). The overall stone-free rate was 78.8% and perioperative complication was observed in nine patients (17.3%). Conservative treatment was undergone by 22 patients (29.7%) (conservative management group). Two-year overall survival rates in surgical treatment and conservative management groups were 88.0% and 38.4%, respectively (*p* < 0.01) and two-year stone-specific survival rates in the two groups were 100.0% and 61.3%, respectively (p < 0.01). On multivariate analysis, stone removal was not significant, but was considered a possible favorable predictor for overall survival (*p* = 0.07). Moreover, stone removal was the only independent predictor of stone-specific survival (p < 0.01).

**Conclusions:**

Active stone removal for patients with poor performance status could be performed safely and effectively. Compared to conservative management, surgical stone treatment achieved longer overall survival and stone-specific survival.

**Electronic supplementary material:**

The online version of this article (10.1186/s12894-017-0293-4) contains supplementary material, which is available to authorized users.

## Background

The rate of aging (65 years of age or older) worldwide is expected to rise from 7.6% in 2010 to 18.3% in 2060 as the population increases [1]. As the rate of aging rises, patients with poor performance status (PS) are also expected to increase worldwide. These patients have increased risk of urolithiasis because of various factors, including hypercalciuria associated with osteoporosis, urinary stasis, urinary tract infection and low fluid intake. Therefore, management of urolithiasis in patients with poor PS is emerging as a crucial issue in urology.

However, debate exists as to whether active stone removal should be performed in patients with poor PS. One of the reasons for such debate is that patients with poor PS have poor prognoses because of their comorbidities [2–4]. In addition, active stone removal for poor PS patients is introduced to deal with various problems, such as risks involved in their comorbidities, decreased immune competence, coexisting urinary tract infection and restriction on surgical positioning. Nonetheless, it is necessary to investigate whether active stone removal for patients with poor PS is beneficial.

To date, there have been few reports concerning the optimal management of urolithiasis in these patients. In this study, we evaluate treatment outcomes of active stone removal in patients with poor PS and compared life prognosis with those managed conservatively.

## Methods

### Patients

Between January 2009 and March 2016, 81 patients with Eastern Cooperative Oncology Group (ECOG) PS 3 or 4 were hospitalized for upper urinary tract calculi at the Wakayama Medical University Hospital, Hashimoto Municipal Hospital, Kinan Hospital and Rinku General Medical Center. Of these, seven patients who experienced spontaneous stone expulsion were excluded and 74 patients were enrolled in this study. This retrospective study was approved by the Institutional Review Board of Wakayama Medical University (approval number 1922).

Clinical information including age, gender, ECOG PS, medical history, Charlson Comorbidity Index (CCI), serum creatinine on admission, coexisting acute pyelonephritis, urinary drainage and stone characteristics were collected retrospectively from medical records. Urinary drainage was defined as placement of ureteral stent or nephrostomy tube on admission. Stone characteristics, including size, location and number, were assessed by non-contrast computed tomography (NCCT) and stone size was defined as the largest diameter of the major stone. In addition, data were collected on the management of any stones, such as active stone removal, including shock wave lithotripsy (SWL), ureteroscopy (URS) and percutaneous nephrolithotomy (PCNL), and observation without operation. Patients were classified into two groups, surgical treatment group and conservative management group, based on occurrence of active stone removal. The treatment policy was left to the judgment of attending physicians, patients and their families.

### Outcomes

We investigated the stone-free rate (SFR) and perioperative complications (Clavien-Dindo system grade II or more) in the operation group. Stone-free status was determined using NCCT within 3 months after operation and was defined as the absence of stones or residual fragments of less than 4 mm. We also investigated stone-related event-free survival (EFS), with events defined as stone-related symptoms and interventions, and recurrence-free survival (RFS), with recurrence defined as new stone formation and/or regrowth of residual fragments on imaging studies as well as any stone-related events. In addition, we defined stone-specific survival (SSS) as a net survival measure representing urolithiasis survival in the absence of other causes of death, and compared overall survival (OS) and SSS between the surgical treatment group and the conservative management group.

### Statistical analyses

Chi-square test, Fisher’s exact test and Mann-Whitney U test were used for univariate analyses to compare variables between surgical treatment group and conservative management group. EFS, RFS, OS and SSS rates were calculated by the Kaplan-Meier method with the hospitalization date as the starting point. Univariate and multivariate analyses of OS and SSS were performed to compare the prognostic factors in a Cox proportional hazards analysis. Any *P* values less than 0.05 were considered significant. All statistical analyses were performed using JMP Pro 12 (SAS Institute, USA).

## Results

### Patient demographics

Patient demographics and stone characteristics are summarized in Table 1. The median age was 82 years (range: 36–98 years) and 51 (68.9%) patients were females. ECOG PS was 3 in 19 (25.7%) patients and 4 in 55 (74.3%) patients. Sixty-one (82.4%) patients presented acute pyelonephritis. The median stone size was 11.5 mm (range: 2–53 mm). More information about patient demographics and stone characteristics can be found in Additional file 1: Table S1.

**Table 1:** Patient demographics

	Total	Management		*p* value (surgical treatment vs. conservative management)
		Surgical treatment	Conservative management	
No. patients (%)	74	52 (70.3)	22 (29.7)	
Age, years	82 (36–98)	76 (36–92)	86 (68–98)	<0.01
Female, n (%)	51 (68.9)	33 (63.5)	18 (81.8)	0.17
ECOG PS, n (%)	19 (25.7)	16 (30.8)	3 (13.6)	0.15
3	55 (74.3)	36 (69.2)	19 (86.4)	
4				
Charlson comorbidity index	2 (0–8)	2 (0–6)	2 (0–8)	0.82
Serum creainine on admission, mg/dL	1.01 (0.33–5.49)	0.89 (0.44–5.49)	1.26 (0.33–3.83)	0.28
History of urinary calculi, n (%)	20 (27.0)	16 (30.8)	4 (18.2)	0.39
Coexisting acute pyelonephritis, n (%)	61 (82.4)	40 (76.9)	21 (95.5)	0.09
Urinary drainage	61 (82.4)	43 (82.7)	18 (81.8)	1.00
Bilateral stone, n (%)	7 (9.5)	7 (13.5)	0 (0.0)	0.09
Stone position, n (%)				
Kidney	17 (23.0)	14 (26.9)	3 (13.6)	
Ureter	44 (59.5)	28 (53.9)	16 (72.7)	
Kidney and ureter	13 (17.6)	10 (19.2)	3 (13.6)	
Stone size, mm	11.5 (2–53)	12 (2–53)	10.5 (4–35)	0.82
Multiple stones, n (%)	41 (55.4)	28 (53.9)	13 (59.1)	0.79

*Abbreviations: ECOG*, Eastern Cooperative Oncology Group, *PS* performance status

Continuous variables are shown in “median (range)” form

Of 74 patients included in this study, 22 patients (29.7%) did not undergo stone removal and were classified as conservative management group. In conservative management group, 15 patients (68.2%), three patients (13.6%) and four patients (18.2%) had a ureteral stent, a nephrostomy tube and neither of these, respectively. A further 52 (70.3%) patients underwent active stone removal and were classified as surgical treatment group patients. The median interval between first admission and definitive therapy in surgical treatment group was 41 days (range: 2–243 days). The patients with acute pyelonephritis at admission underwent surgical treatment after improvement of infections. Comparing patients in surgical treatment group and those in conservative management group, the median age of patients in conservative management group was 86 years and significantly older than in surgical treatment group (*p* < 0.01). Coexisting acute pyelonephritis and unilateral stones seemed to be more frequently observed in conservative management group compared with surgical treatment group (*p* = 0.09 and p = 0.09, respectively).

### Treatment outcomes in surgical treatment group

Patients in operation group were treated by SWL (*n* = 6, 8.8%), URS (*n* = 39, 52.7%), PCNL (*n* = 6, 8.1%) or nephrectomy (*n* = 1, 1.4%). All PCNL cases were performed in a prone split-leg position and combined with retrograde flexible ureteroscopy. Overall SFR in operation group was 78.8% and by the operative treatment method, 50.0% in SWL, 87.2% in URS and 50.0% in PCNL (Table 2). Nine patients (17.3%) experienced perioperative complications. Postoperative pyelonephritis (Clavien-Dindo system grade II) were observed in eight patients (one out of six SWL patients, six out of 39 URS patients and one out of six PCNL patients) and other complication, namely pseudoaneurysm (Clavien-Dindo system grade III), was observed in one out of six PCNL patients (Table 2).

**Table 2:** Stone-free rates and perioperative complications in operation group

	No. pts.	Stone-free pts., *n* = (%)	Perioperative complications, *n* = (%)		
			Postoperative pyelonephritis	Others	Total
SWL	6	3 (50.0)	1 (16.7)	0 (0.0)	1 (16.7)
URS	39	34 (87.2)	6 (15.4)	0 (0.0)	6 (15.4)
PCNL	6	3 (50.0)	1 (16.7)	1 (16.7)	2 (33.3)
Nephrectomy	1	1 (100.0)	0 (0.0)	0 (0.0)	0 (0.0)
Total	52	41 (78.8)	8 (15.4)	1 (1.9)	9 (17.3%)

**Fig. 1:**
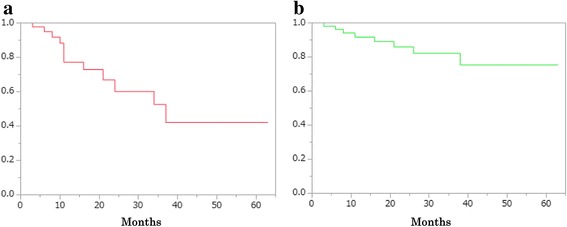
**a** Stone recurrence-free survival rate and **b** Stone-related event-free survival rate in operation group

Two-year and five-year RFS rates in surgical treatment group were 60.2% and 42.1%, respectively (Fig. 1a). On the other hand, two-year and five-year EFS rates were 86.0% and 75.4%, respectively (Fig. 1b).

### Comparison of overall survival and stone-specific survival rates between surgical treatment group and conservative management group

**Fig. 2:**
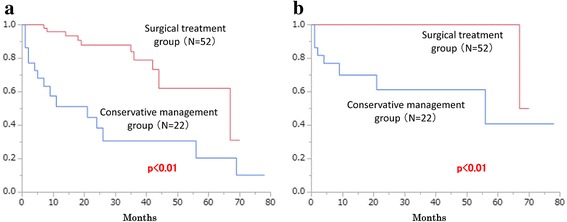
Comparison of **a** overall survival rate and **b** stone-specific survival rate between operation group and non-operation group

In entire cohort, nine patients (12.2%) died of pyelonephritis or renal failure associated with urolithiasis and 17 patients (23.0%) died of other causes during the observation period (median 23 months, range: 1–78 months). A total of 15 patients in conservative management group died and, of these patients, 12 patients (80.0%), 1 patient (6.7%) and 2 patients (13.3%) had a ureteral stent, a nephrostomy tube and neither of these, respectively. Two-year OS rates in surgical treatment and conservative management groups were 88.0% and 38.4%, respectively (*p* < 0.01, Fig. 2a), while two-year SSS rates in surgical treatment and conservative management groups were 100.0 and 61.3% (*p* < 0.01, Fig. 2b).

### Associations between various parameters and overall survival

Univariate and multivariate Cox proportional hazards regression models were used to investigate predictors of OS (Table 3). Among several predictors, age (*p* < 0.01), female (*p* = 0.02) and stone removal (p < 0.01) were identified as significant predictors for OS on univariate analysis. Furthermore, CCI trended toward significance (*p* = 0.06). Of these factors, age (HR 1.08, 95% CI 1.02–1.15) and CCI (HR 1.36, 95% CI 1.10–1.68) were independent unfavorable predictors of OS on multivariate analysis. Stone removal was not significant, but was considered a possible favorable predictor of OS (HR 0.43, 95% CI 0.16–1.09).

**Table 3:** Univariate and multivariate analyses of associations between various parameters and overall survival

Variable	Univariate analysis			Multivariate analysis		
	HR	95% CI	*p* value	HR	95% CI	*p* value
Age, year	1.09	1.05–1.15	<0.01	1.08	1.02–1.15	<0.01
Female (vs Male)	3.10	1.17–10.70	0.02	1.92	0.63–7.41	0.26
Charlson Comorbidity Index	1.28	0.98–1.64	0.06	1.36	1.10–1.68	<0.01
Coexisting acute pyelonephritis	1.53	0.58–5.26	0.42			
Urinary drainage	0.94	0.38–2.83	0.90			
Stone size, mm	0.99	0.94–1.03	0.52			
Multiple stones	0.89	0.41–1.96	0.75			
Stone removal	0.22	0.10–0.49	<0.01	0.43	0.16–1.09	0.07

### Associations between various parameters and stone-specific survival

Table 4 shows the results of univariate and multivariate Cox proportional hazards regression models of factors which predict SSS. Age (p < 0.01) and stone removal (p < 0.01) were significantly associated with SSS in univariate analysis. Female gender trended toward significance (*p* = 0.08). Of the factors, stone removal was the only independent predictor of SSS in multivariate analysis (HR 0.06, 95% CI 0.00–0.43).

**Table 4:** Univariate and multivariate analyses of associations between various parameters and stone-specific survival

Variable	Univariate analysis			Multivariate analysis		
	HR	95% CI	p value	HR	95% CI	p value
Age, year	1.10	1.02–1.22	<0.01	1.01	0.91–1.14	0.90
Female (vs Male)	4.59	0.83–85.69	0.08	3.80	0.39–95.67	0.27
Charlson Comorbidity Index	1.21	0.79–1.78	0.36			
Coexisting acute pyelonephritis	1.99	0.36–37.98	0.47			
Urinary drainage	0.43	0.11–2.07	0.26			
Stone size, mm	0.97	0.86–1.04	0.45			
Multiple stones	1.55	0.40–7.39	0.53			
Stone removal	0.05	0.00–0.27	<0.01	0.06	0.00–0.43	<0.01

## Discussion

We analyzed the treatment outcomes of active stone removal in patients with poor PS and compared life prognosis with those managed conservatively. In this study, we made three important clinical observations.

First, if patients who are suitable for active stone removal are appropriately selected, active stone removal could be performed safely, in spite of their poor PS. One of the main reasons for avoiding surgical treatment for patients with poor PS is the risk of perioperative complications, especially infectious disease. A review of the current literature on the management of urolithiasis in patients with spinal cord injury showed that the overall complication rate in patients with spinal cord injury is higher than in the general population, and the majority of these are infectious in nature are due to the associated medical comorbidities and chronic bacteriuria [5]. In our study, postoperative pyelonephritis was observed in 16.7% (1/6 cases), 15.4% (6/39 cases) and 16.7% (1/6 cases) of the patients who underwent SWL, URS and PCNL, respectively. This was higher than previously reported [6–9]. However, major perioperative complications (Clavien-Dindo system grade III or more) were observed in only one patient and no patients expired perioperatively. These results suggest that perioperative complications were acceptable, given the comorbidities in patients with poor PS. In spite of this, these results do not necessarily mean that active stone removal, even for patients in conservative management group, could be performed safely. Patient backgrounds are different between surgical treatment and conservative management groups. We suggest that active stone removal for patients with poor PS could be performed safely as long as perioperative risks are assessed and managed carefully.

Second, results of the survival analyses suggested that patients who had active interventions achieved longer survival in spite of their poor PS. Another reason why physicians choose conservative treatment for patients with poor PS is the assumption that they have poor prognoses because of the multiple comorbidities. Slot et al. reported that the median survival of patients with poor PS resulting from ischaemic stroke was 2.5 years after the stroke [3]. Xie et al. reported that the median survival of patients with dementia was 4.5 years after the onset [2]. Hossain et al. reported that approximately one in five people with spinal cord injury who are wheelchair-dependent die within 2 years of discharge from hospital [10]. Considering the results of these studies, it is questionable whether active stone removal is recommended for patients with poor PS. However, to our knowledge, there are no published studies related to the influence of stone removal on patients with poor PS.

In the present study, the two-year OS rate in the surgical treatment group (88.0%) was better than that in the conservative management group (38.4%). Multivariate Cox proportional hazards regression model shows that stone removal is the only independent predictor of SSS. In addition, stone removal is not significant, but is considered a possible favorable predictor of OS. These survival analyses showed that the patients who had active interventions achieved their longer survival in spite of their poor PS. However, these results do not necessarily suggest that active stone removal in patients with poor PS could prevent stone-related death and may improve their prognosis, because patient characteristics are different between two groups. The median age of patients in the conservative management group was 86 years, significantly older than that in the surgical treatment group (*p* < 0.01). Moreover, preoperative pyelonephritis seemed to be more frequently observed in the conservative management group compared with the surgical treatment group (*p* = 0.09). Although we adjusted the patient backgrounds using various factors, such as age, sex, coexisting acute pyelonephritis and CCI, as possible in multivariate Cox proportional hazards regression model, other differences which are not reflected in these factors could have a strong influence on the patients’ prognosis. Therefore, randomized controlled trials or a large-scale propensity score matching analysis using even more factors are necessary to make clear whether active stone removal in patients with poor PS could prevent stone-related death and could improve their prognosis.

Third, active stone removal for patients with poor PS could prevent stone-related events. In the present study, overall SFR in the surgical treatment group was 78.8%. This is acceptable considering the adverse conditions specific to patients with poor PS. However, when new stone formation and regrowth of residual fragments were included in the definition of recurrence, two-year and five-year RFS rates were low at 60.2% and 42.1%, respectively. This might be because of risks of stone formation specific to patients with poor PS such as hypercalciuria associated with osteoporosis, urinary stasis, urinary tract infection and low fluid intake. In addition, prevention of stone recurrence is also difficult in these patients because of decreased accessibility to medical services and compliance to fluid intake and medication. However, given the fact that their life expectancy is generally short, we believe that it is more important to prevent stone-related symptoms and avoid further interventions for stones, rather than preventing radiographic recurrence. From that point of view, two-year EFS rate of 86.0% and five-year EFS rate of 75.4% were considered to be satisfactory.

To reduce the recurrence rate, the achievement of stone-free status is important. In our study, by the operative treatment method, SFR was 50.0% in SWL, 87.2% in URS and 50.0% in PCNL, respectively. The main cause of the low SFR in PCNL cases was considered to be larger stone size (median 27.5 mm, range 9–53 mm) compared with other operations. On the other hand, the reasons for the low SFR in SWL might include the difficulty of spontaneous expulsion of fragments after lithotripsy because of low fluid intake and physical activities in patients with poor PS. Therefore, our results suggest that URS is preferable to SWL in the treatment of patients with poor PS.

There are several limitations to the present study. First, this was a retrospective study undertaken at several centers with a relatively small number of patients. Second, treatment policy was left to the judgment of attending physicians, patients and their families. In addition, this study targeted only hospitalized patients and did not include most patients with asymptomatic calculi.

Despite these limitations in our study, we were able to demonstrate that active stone removal for patients with poor PS could be performed safely and the patients who had active interventions achieved their longer survival with infrequent stone-related events as long as perioperative risks are assessed and managed carefully. To establish the guideline for the optimal management of urolithiasis in these patients, further prospective analysis involving a multicenter approach is required. In addition, an effective method to prevent stone recurrence is essential as it has been a challenge particularly for the patients with poor PS.

## Conclusions

Active stone removal for patients with poor PS could be performed safely and effectively. Compared to conservative management, surgical stone treatment achieved longer OS and SSS.

## Additional file

Additional file 1: Table S1. The demographics and stone characteristics of patients, de-identified raw data. (XLSX 23 kb)

## Abbreviations

CCI: Charlson Comorbidity Index
ECOG: Eastern Cooperative Oncology Group
EFS: Stone-related event-free survival
NCCT: Non-contrast computed tomography
OS: Overall survival
PCNL: Percutaneous nephrolithotomy
PS: Performance status
RFS: Recurrence-free survival
SFR: Stone-free rate
SSS: Stone-specific survival
SWL: Shock wave lithotripsy
URS: Ureteroscopy
